# Supplementary material for: Conservation of the glycogen metabolism pathway underlines a pivotal function of storage polysaccharides in Chlamydiae
Source: Commun Biol. 2021 Mar 5;4:296. doi: 10.1038/s42003-021-01794-y (PMC7935935; doi:10.1038/s42003-021-01794-y)
Supplement: Supplementary file 1 — Supplementary Information [file 42003_2021_1794_MOESM1_ESM.pdf]

SUPPLEMENTARY INFORMATION

Conservation of the glycogen metabolism pathway underlines a pivotal function of storage polysaccharides in *Chlamydiae*

Matthieu Colpaert[1], Derifa Kadouche[1b], Mathieu Ducatez[1b], Trestan Pillonel[2], Carole Kebbi-Beghdadi[2], Ugo Cenci[1], Binquan Huang[1c], Malika Chabi[1], Emmanuel Maes[1], Bernadette Coddeville[1], Loïc Couderc[3], Hélène Touzet[4], Fabrice Bray[5], Catherine Tirtiaux[1], Steven Ball[1], Gilbert Greub[2], Colleoni Christophe[1*].

Supplementary Table 1: Organization of *glg* genes of GlgC pathway across Chlamydial genomes.

Supplementary Table 2: List of pairs of primers used for cloning the gene involved in glycogen metabolism pathway.

Supplementary Figure 1: Domain organization of fused protein GlgA-GlgB and proton-NMR analyses of incubation products.

Supplementary Figure 2: Characterization of both recombinant proteins GlgE of *Estrella Lausannensis* and *Waddlia chondrophila*

Supplementary Figure 3: Proton- and phosphate-NMR analyses of maltose-1-phosphate.

Supplementary Figure 4: FACE analysis of activity GlgE of *Estrella Lausannensis.*

Supplementary Figure 5: FACE analysis of activity GlgE of *Waddlia chondrophila.*

Supplementary Figure 6: Native-PAGE analysis of GlgE of *E. lausannensis* and branching enzyme activity of *Waddlia chondrophila*.

Supplementary Figure 7: Characterization of recombinant his-tag TreS-Mak.

Supplementary Figure 8: Heterologous secretion assay in *Shigella flexneri* of GlgE and TreS-Mak proteins.

Genome accession	species	organization of glg genes				
NZ_LT999999	*Chlamydia suis isolate 4-29b*	X	P	C	A	B
CP000051	*Chlamydia trachomatis A/HAR-13*	X	P	C	A	B
AM884177	*Chlamydia trachomatis L2b/UCH-1/proctitis*	A	B	X	P	C
CP021996	*Chlamydia abortus GN6*	C	B	X	P	A
CP001713	*Chlamydia pneumoniae LPCoLN*	C	B	X	P	A
AE009440	*Chlamydia pneumoniae TW-183*	P	X	B	C	A
CR848038	*Chlamydia abortus S26/3*	C	B	X	P	A
AE015925	*Chlamydia caviae GPIC*	C	B	X	P	A
CP002549	*Chlamydia psittaci 6BC*	C	B	X	P	A
CP004033	*Chlamydia pecorum PV3056/3*	C	B	X	P	A
APJW01000000	*Chlamydia ibidis 10-1398/6*	C	B	X	P	A
ATNB01000000	*Chlamydia ibidis 10_1398_11*	X	P	A	C	B
MKSK01000000	*Chlamydiales bacterium 38-26*	A	B	X	C	P
CP019792	*Chlamydia gallinacea JX-1*	P	X	B	C	A
AE002160	*Chlamydia muridarum Nigg*	A	B	X	P	C
AP006861	*Chlamydia felis Fe/C-56 Fe/C-56*	A	P	X	B	C
CP002608	*Chlamydia pecorum E58*	A	C	B	X	P
CP015840	*Chlamydia gallinacea 08-1274/3*	A	C	B	X	P
CP006571	*Chlamydia avium 10DC88*	B	X	P	A	C
FR872580	*Parachlamydia acanthamoebae UV-7*	C	P	X	B	A
ACZE01000000	*Parachlamydia acanthamoebae Hall's coccus*	C	P	B	A	X
JSAM01000000	*Parachlamydia acanthamoebae OEW1*	C	P	X	B	A
AP017977	*Neochlamydia sp. S13*	A	X	B	P	C
JSAN01000000	*Candidatus Protochlamydia amoebophila EI2*	P	C	A	B	X
LN879502	*Candidatus Protochlamydia naegleriophila KNic*	X	B	A	C	P
DNHV01000000	*Parachlamydiales bacterium*	X	B	A	C	P
NZ_FCNU00000000	*Parachlamydia sp. C2*	B	X	A	C	P
CAAJGQ000000000	*Rhabdochlamydia sp. T3358*	X	P	A	C	B
NZ_CCJF00000000	*Protochlamydia massiliensis*	A	C	P	X	B
NZ_BAWW00000000	*Parachlamydia acanthamoebae Bn9*	P	B	A	C	X
FR872582	*Simkania negevensis Z main*	C	P	B	X	A

Supplementary Table 1: Organization of *glg* genes of GlgC pathway across Chlamydial genomes. Genes encoding for ADP-glucose pyrophosphorylase (*glgC*); glycogen synthase (*glgA*), glycogen branching enzyme (*glgB*), glycogen phosphorylase (*glgP*) and glycogen debranching enzyme were listed according to their organization on chlamydial genomes. With a notable exception for *glgC* and *glgP* genes, which are often separated by one or two genes, most of *glg* genes are encoded more than 10 kpb from each other.

Name	Sequence	Hybridation temperature	Destination vector	Accesion number
F_glgE_EL<br>R_glgE_EL	GGGGACAAGTTTGTACAAAAAAGCAGGCTTCATGATGAGTGTTTCTGCTGCCGAA<br>GGGGACCACTTTGTACAAGAAAGCTGGGTCTCATATAAAGTAATCGAAATTGTTTTCCCTCAGCATC	56,9 °C	pDONR 221	WP_098038073.1
F_glgE_WC<br>R_glgE_WC	GGGGACAAGTTTGTACAAAAAAGCAGGCTTCATGGGACAAAATCGAGTCGTCA<br>GGGGACCACTTTGTACAAGAAAGCTGGGTCTCACATGAAATAGTCGAATTGTTG	61,5 °C	pDONR 221	WP_013182917.1
F_treS-mak_EL<br>R_treS-mak_EL	GGGGACAAGTTTGTACAAAAAAGCAGGCTTCATGAAACAAGATCCGCTCTGGT<br>GGGGACCACTTTGTACAAGAAAGCTGGGTCTTAACGCATCTCCTTGAGGATATT	56,0 °C	pDONR 221	WP_098038072.1
F_glgB_WC<br>R_glgB_WC	GGGGACAAGTTTGTACAAAAAAGCAGGCTTCATGAAAGAAGCAGTCGCCGTT<br>GGGGACCACTTTGTACAAGAAAGCTGGGTCCTATTCCGGGGACAGACAAA	56,9 °C	pDONR 221	WP_013182919.1
F_glgA_Ecoli<br>R_glgA_Ecoli	GGGGACAAGTTTGTACAAAAAAGCAGGCTTCATGATGCAGGTTTTACATGTATGTTCAG<br>GGGGACCACTTTGTACAAGAAAGCTGGGTCCTATTTCAAGCGATAGTAAAGCTCA	59,8 °C	pDONR 221	CP009273.1
F_glgA-glgB_EL<br>R_glgA-glgB_EL	GGGGACAAGTTTGTACAAAAAAGCAGGCTCGATGAAAATATTTAATATTGAAAGCAAATG<br>GGGGACCACTTTGTACAAGAAAGCTGGGTATTAGCCAAAGATTTTTTTTGATG	56,9 °C	pDONR 221	WP_098037869.1
F_glgA-glgB_WC<br>R_glgA-glgB_WC	GGGGACAAGTTTGTACAAAAAAGCAGGCTCGATGACCGTTTCCGCCATT<br>GGGGACCACTTTGTACAAGAAAGCTGGGTACTAACCATGATGGACCTCCT	61,0 °C	pDONR 221	WP_013181537.1
F_sec_glgE_EL<br>R_sec_glgE_EL	AGTCAAGCTTGTAATAGTTTTGTTTTTATGAGTGTTTCTGCTGCCGAAAAG<br>AGTCTCTAGAAACGATAGAGTGCAAAGTATTGGTATACAT	59,8 °C	pUC19cya	WP_098038073.1
F_sec_glgE_WC<br>R_sec_glgE_WC	AGTCAAGCTTGTAATAGTTTTGTTTTTATGGGACAAAATCGAGTCGTCA<br>AGTCTCTAGAGATAGACTCTCCTAAGGTTCGTT	58,0 °C	pUC19cya	WP_013182917.1
F_sec_treS-mak_EL<br>R_sec_treS-mak_EL	AGTCAAGCTTGTAATAGTTTTGTTTTTATGAAACAAGATCCGCTCTGGTTTAAACACG<br>AGTCTCTAGATCCGATGCCGTCGTGATTG	59,8 °C	pUC19cya	WP_098038072.1
F_sec_treS-mak_WC<br>R_sec_treS-mak_WC	AGTCAAGCTTGTAATAGTTTTGTTTTTATGATGCACAACTGGTATAAAGACGCAAT<br>AGTCTCTAGAGAAATCGCCGATGCCGTCTT	59,8°C	pUC19cya	WP_013182919.1

Supplementary Table 2: List of pairs of primers used for cloning the genes involved in glycogen metabolism pathway of *E. lausannensis* and *W. chondrophila* and for heterologous secretion assay. Underlined nucleotides represent the attB sites added to the amplified genes that allow the cloning into pDONR 221 vectors following the recommendation of Thermofisher (Gateway^{TM}).

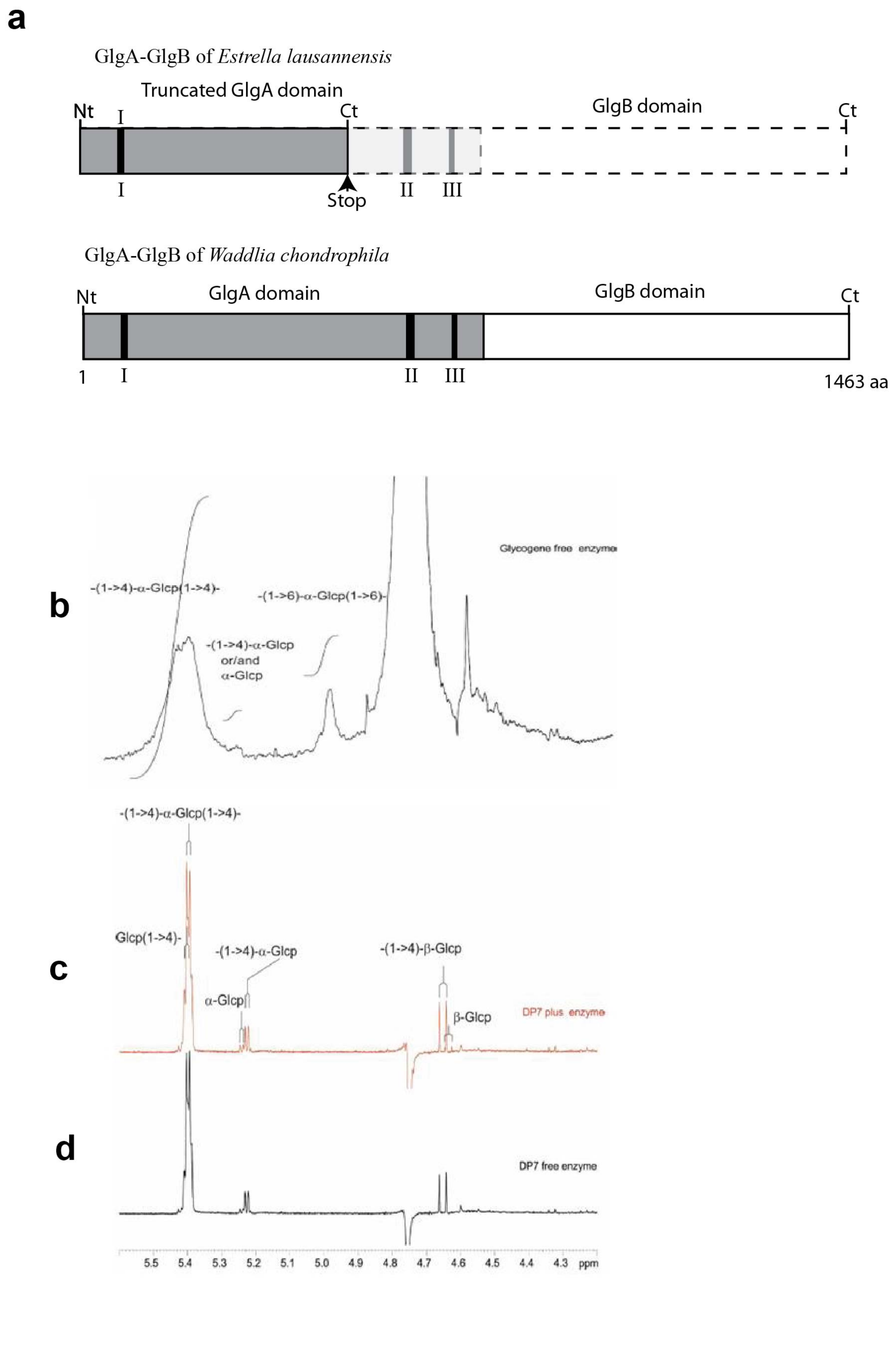

Supplementary Figure 1: **a** Domain organization of fused protein GlgA-GlgB of *E. lausannensis* and *W. chondrophila*. Glycogen synthase domain (gray box) and branching enzyme domain (white box) are respectively located at the N- (Nt) and C-termini (Ct) respectively. The insertion of one-nucleotide in *E. lausannensis* sequence results in a frame shift and the appearance of truncated GlgA-GlgB protein. Regions I, II and III represent

highly conserved sequences in the glycogen synthase GT5 family that includes amino acid residues involved in the catalytic site and nucleotide binding sites. **b** Proton-NMR analyses of glycogen from rabbit liver and maltoheptaose (10 $mg.mL^{-1}$) + ADP-glucose (3 mM) incubated overnight at 30°C in the presence (**c**) or in the absence (**d**) of recombinant GlgA-GlgB fusion enzyme of *Waddlia Chondrophila.* After incubation, enzymatic reactions were boiled and purified through anion and cation exchange resins (DOWEX 1 X 8 and DOWEX 50 W X 8). Protons involved in α-1,4 linkages or α-1,6 linkages resonate at 5.4 and 4.95 ppm, respectively. Protons in α and β position on C1 (reducing end) generate signals at 5.23 and 4.65 ppm. The absence of signal at 4.95 ppm suggests that either signal corresponding to α-1,6 linkages is below threshold of detection (<1%) or GlgB domain is not active in the GlgA-GlgB of *W. chondrophila*.

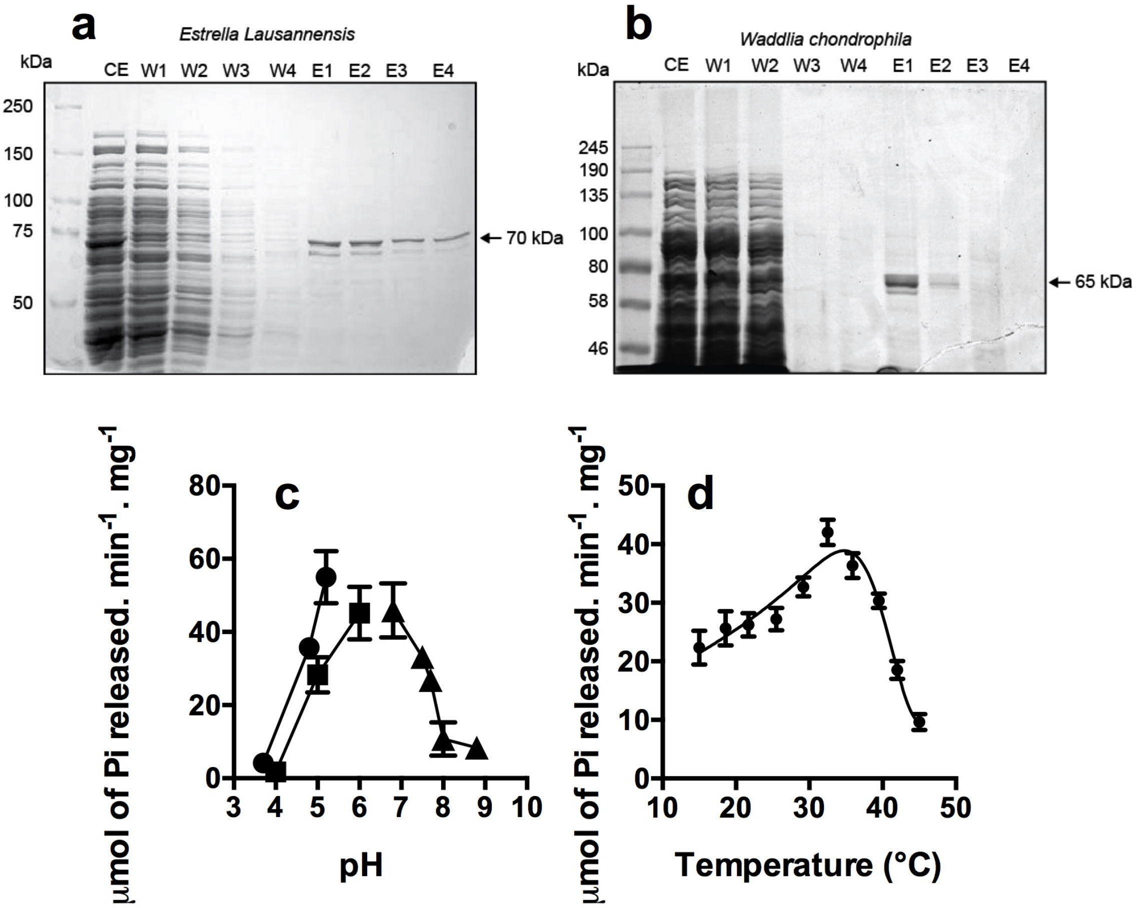

e

GlgE_EL Uniprot accession number : A0A0H5DRC6

MGSSHHHHHH SSGLVPRGSH MLEDHQTSLY KKAGFMMSVS AAEKRQLLTQ VKASGSMYTN TLHSIVIQNI SPKPEPFGPA
KRVLLEQVEV EADIFTDPHT KLEADLIVKA PGLRKTTRIP FHPLVNDRFK AVFVPDTPGT YSFFVEAWVN PAALWQEDLR
KKQEARLDLT VDLQIGAALL VEVADLLPPE ESVKLLQIAK KLREQKGAEA VAFDPEIARL GRLARPKSRQ GVCSQEQEVF
VTRKKALFST WYELFPRSCS GTMRHGTFND VVAKLPRISR MGFDVLYLPP IHPIGKTKRK GKNNALTALP DDPGSPWAVG
SHEGGHDAIQ RELGTLADFE RLVKEAESLG IEIALDIALQ CSPDHPYLKE HPEWFLKRPD GTYQYAENPP KKYEDIIPFH
FTEDNKDTLF REILRIFLFW MKRGVRIFRV DNPHTKPFAL WEYVIGEVRK VDSGVIFLAE AFTRPKIMHH LARIGFDQSY
TYFTWRNTKW ELTEYLTELT KTEARNYLRP NFFVNTPDIL PESLQWGGRA AFLTRLILAA TLSSSYGIYG PAFEQMLSEA
VPGKEDYIDS EKYEIKPWDF EKKGTISETI EIVNRIRKEN PALQATGNLE FYATDNDYLL AYGKQGPQGE SLLIVVNLDH
YHTQSGYVTL PLMHLGIQEK EPYLVDDLLS GERYIWEGER NYIQLSPHLV PAHIFRIRPK MLRENNFDYF I

GlgE_WC Uniprot accession number : D6YT22

MGSSHHHHHH SSGLVPRGSH MLEDHQTSLY KKAGFMGQNR VVIEHVSPQI NCGQFPIKRT LGESIRISAD IFSDGHDKLY
ANVLWKHKNH RSWKKEKMHF SGNDSWTAEF TPEKTGIYLY TVIAGIDHLS TWYSDLLKKI EASQPIMNDL KTGARLLKRL
SRIKKIEEIL QLIETEKGIE LLKKPQTGKF LSTLFHNYPE EQFLTRYHKE LEMEVSRIKA RFSTWYELFP RSTSKRKGSH
GTFKDCQKLL PEIASMGFDV LYFPPIHPIG IEHRKGKNNQ IKSHKNDPGS PWAIGSKEGG HDAVHPQLGT LEDYRELHKE
AKKFGIELAL DLAFQCSPDH PYIKEHPEWF LWQPDGTVRY AENPPKKYQD IVPFDFECAD WKSLWDELKR IVLFWCKSGV
RIFRVDNPHT KPFAFWQWLI AEVKTDYPDT LFLSEAFTRP KVMQHLAKIG FDQSYTYFTW RNTKKELEDY FQELASSEYL
RPNFWPNTPD ILPQFLQQKK RQAYVARLVL AATASSNYGI YGPCFEYMEH SSLEENSEEY LNSEKYEIRH WEAPKETLKE
LIALVNAIRK GNAALQFTRN LTIIPNDNPQ IISYAKQSPI NDNLLMIVVC LDPQHTQSGW LSIPSEQLGL PTDQPYLVQD
LLSGDKYMWQ GNSAFIELNP FVIPAHIFKV CKKMIKEQQF DYFM

Supplementary Figure 2: SDS-PAGE analyses of recombinant GlgE after affinity column purification and determination of optima pH and temperature of GlgE-EL**.** Mean and standard deviation of three independent experiments are plotted. **a** His-tag GlgE of *E. lausannensis* and **b** *W. chondrophila* were expressed in Rosetta^{TM} *E. coli* strain. After induction at mid exponential growth with IPTG for GlgE-EL and culture in auto-inductible medium for GlgE-WC, the overnight cultures were harvested by centrifugation. Cell pellets were suspended in loading buffer containing 25 mM TRIS/acetate pH 7.5 and then

subjected to sonication. After centrifugation, crude extract (CE) was incubated with nickel affinity column at 4°C for one hour. Total proteins in both CE and affinity purification fractions; flow-through (FT), washing steps (W1 to W4) and elution (E1 to E4) fractions were separated on SDS-PAGE 7.5%. Based on standard molecular weights, the apparent molecular weights of GlgE were estimated at 76 kDa and 72 kDa for *E. lausannensis* and *W. chondrophila*, respectively. **c** The optima of pH and **d** temperature of GlgE of *E. lausannensis* were determined by measuring the amount of orthophosphate released after the transfer of maltosyl moieties of M1P onto the non-reducing end of glucan chains. The optimum pH determination was carried out at 30°C in sodium acetate ((circle), pH 3.7; 4.8; 5.2), sodium citrate ((square), pH 4; 5; 6) and TRIS/HCl ((triangle) pH 6.8; 7.5; 7.7; 8; 8.8) buffers with a final concentration of 25mM. The optimum pH determination was carried out in the presence of 25 mM TRIS/HCl pH 6.8. **e** The uniprot accession numbers and amino acid sequences of recombinant proteins are displayed for each GlgE proteins. Amino acids underscored correspond to the His-tag and the att site produced during the cloning process in the expression vector.

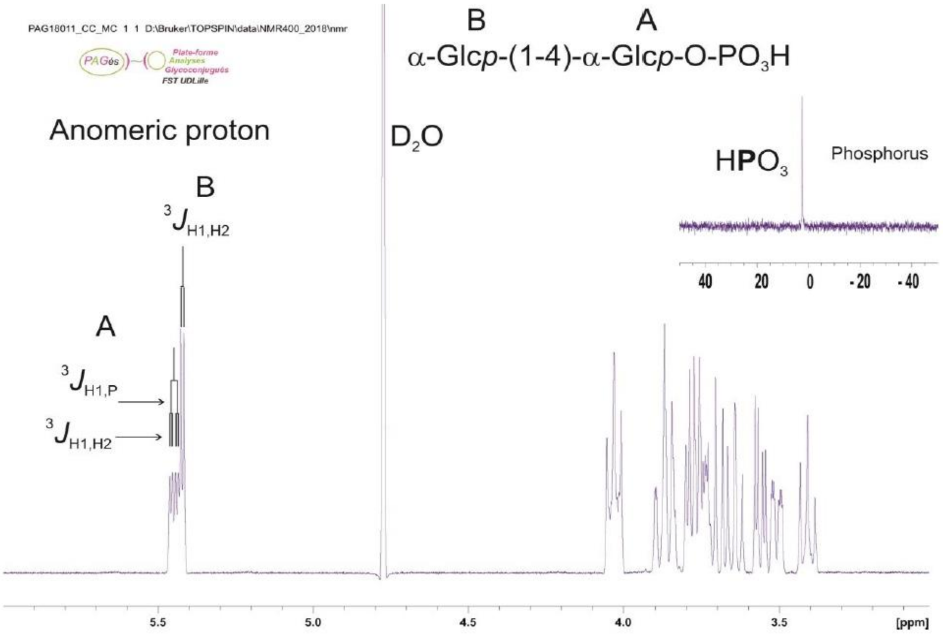

Supplementary Figure 3: Proton- and phosphate-NMR analyses. Reaction product purified following the incubation of *Waddlia chondrophila* GlgE with glycogen and orthophosphate. Complete 1D-^{1}H-NMR spectrum of maltoside-1-phosphate. α-anomer configuration of both glucosyl residues were characterized by their typical homonuclear vicinal coupling constants ($^{3}J_{H1A,H2A}$ and $^{3}J_{H1B,H2B}$) with values of 3.5 Hz and 3.8 Hz respectively. A supplementary coupling constant was observed for α-anomeric proton of residue A as shown the presence of the characteristic doublet of doublet at 5.47 ppm. This supplementary coupling constant is due to the heteronuclear vicinal correlation ($^{3}J_{H1A,P}$) between anomeric proton of residue A and phosphorus atom of a phosphate group, indicating that phosphate group was undoubtedly *O*-linked on the first carbon of the terminal reducing glucosyl unit A. The value of this $^{3}J_{H1A,P}$ was measured to 7.1Hz.

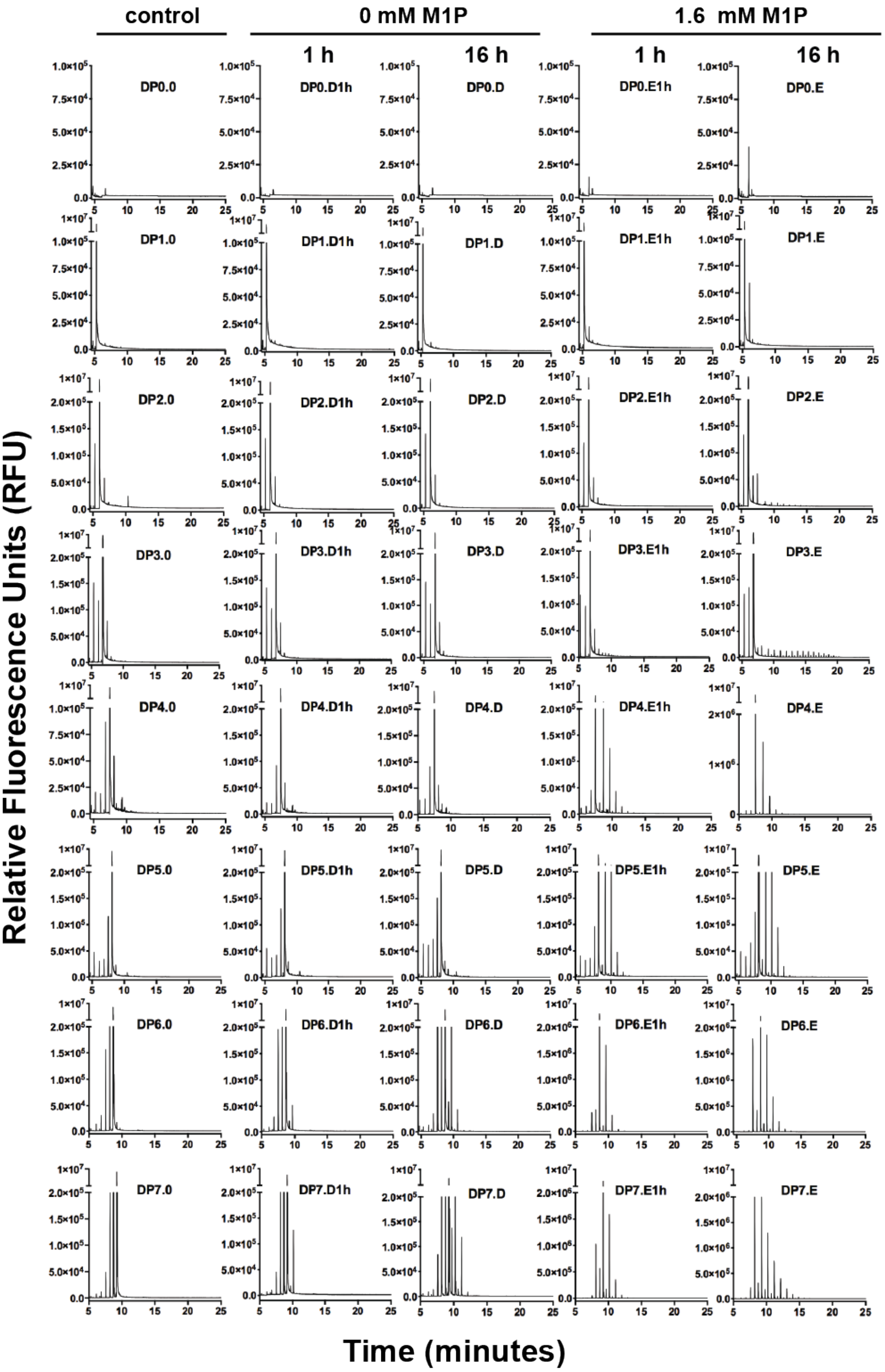

Supplementary Figure 4: FACE analysis of activity GlgE of *Estrella Lausannensis.* Recombinant GlgE (3.5 nmol $Pi.min^{-1}$) was incubated 1 hour and 16 hours at 30°C with 5 mM of malto-oligosaccharides composed of 0 to 7 glucose moieties (degree of polymerization: DP) and 0 mM or 1.6 mM of maltose-1-phosphate (M1P). After incubation, enzymatic reactions are stopped 5 min at 95°C. Malto-oligosaccharides are labeled with APTS and then separated according to their DP using capillary electrophoresis. Fluorescence is monitored as relative fluorescence units (RFU). As control, heat denatured GlgE activity was incubated 16 hours at 30°C with M1P and malto-oligosaccharides.

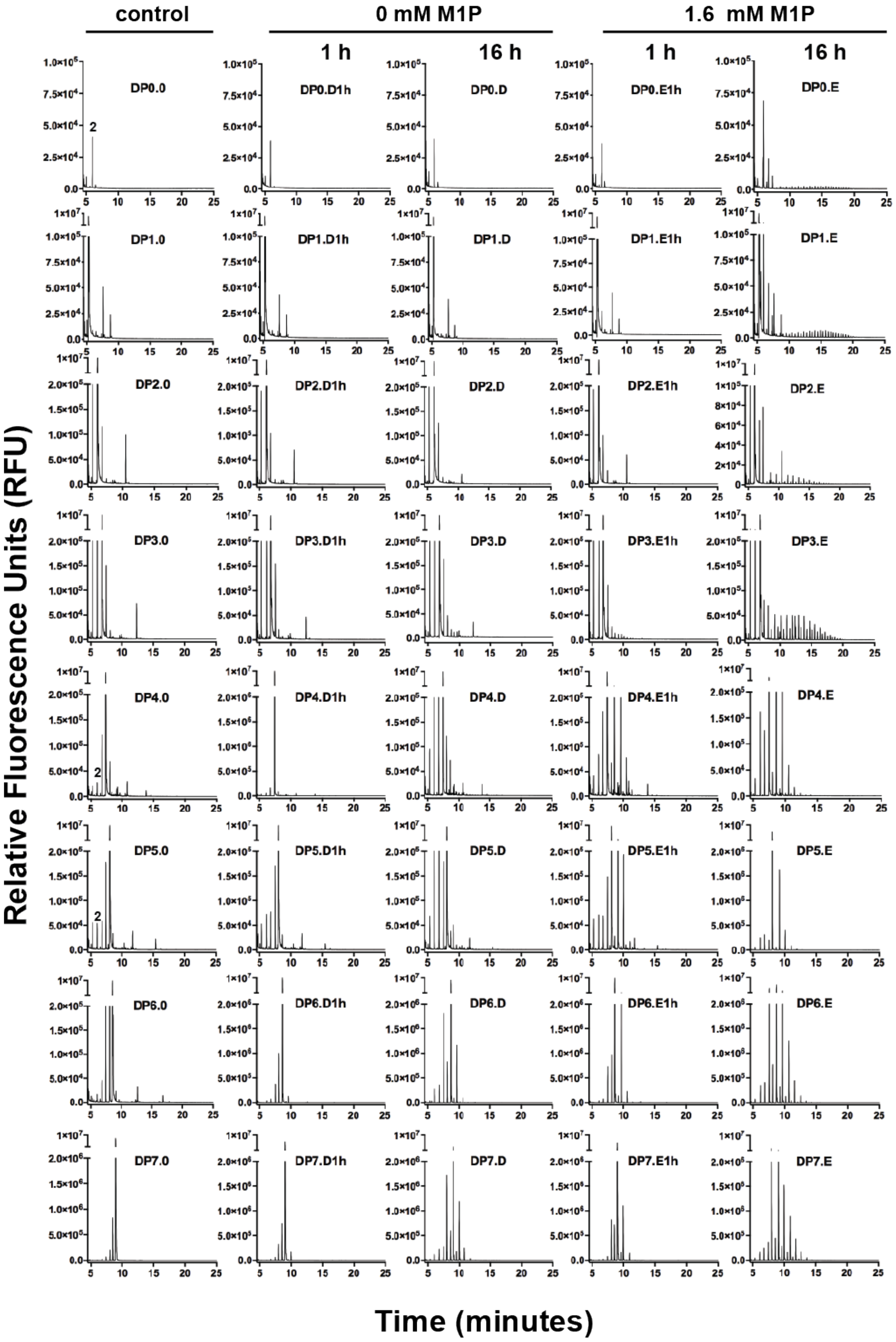

Supplementary Figure 5: FACE analysis of activity GlgE of *Waddlia chondrophila.* Recombinant GlgE (1.38 nmol Pi.min^{-1}) was incubated 1 hour and 16 hours at 30°C with 5 mM of malto-oligosaccharides composed of 0 to 7 glucose moieties (degree of polymerization: DP) and 0 mM or 1.6 mM of maltose-1-phosphate (M1P). After incubation, enzymatic reactions are stopped 5 min at 95°C. Malto-oligosaccharides are labeled with APTS and then separated according to their DP using capillary electrophoresis. Fluorescence is monitored as relative fluorescence units (RFU). As control, heat denatured GlgE activity was incubated 16 hours at 30°C with M1P and malto-oligosaccharides.

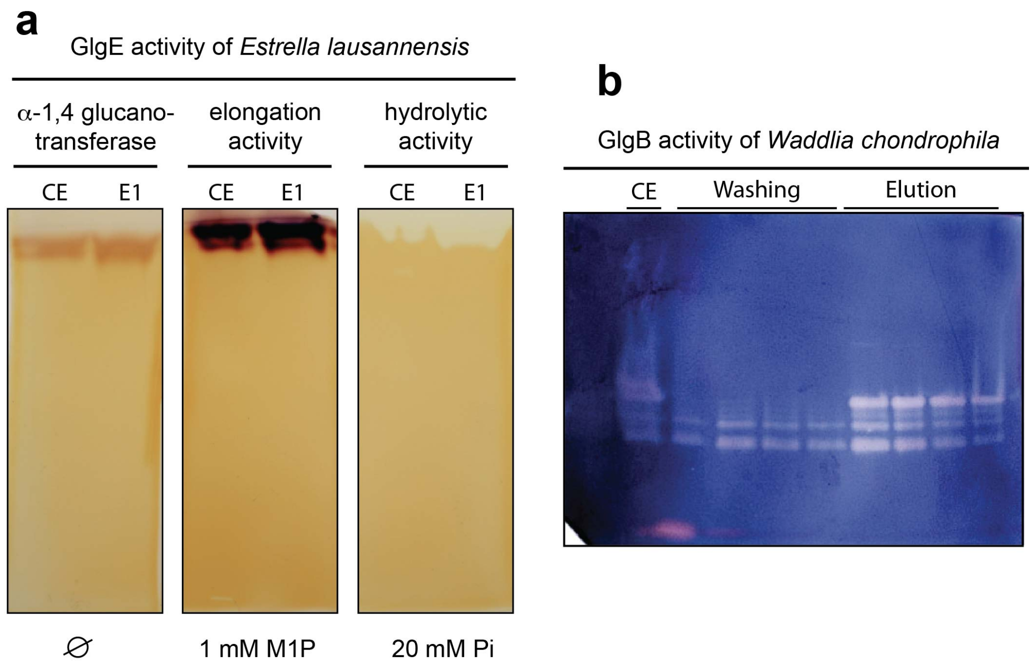

c

GlgB_WC Uniprot accession number : D6YT24

MGSSHHHHHH SSGLVPRGSH MLEDHQTSLY KKAGFMKEAV AVYSLLSEKD IYLFKEGTHD RLYEKLGSHV
VEVDGIKGTQ FAVWAPNAES VSVIGDFNDW DRKRHQLHVR WDESGIWEGF IPGAKQGDCY KYHIASKVNG
YRVEKGDPFA FFAEVPPKTG SIIWDLAYEW KSLTEERSRS DKPISIYEMH FGSWRRDEKN TPPSYREMAE
WLPPYLLDMG FTHVEFMPMM EHPFYGSWGY QKTGYFSPTS RYGSPQDFMF LIESLHQAGI GVFLDWVPSH
FPSDQHGLAF FDGTHLFEHE DPKKGIQPDW NSYIFNYGRN EVRSFLTSSA NFWCSRYHID GLRCDAVASM
LYLDYSRKNG EWIPNAYGGN ENLEAIDFLK TLNTTIYQNH PHVQMIAEES TAWPMVSRPV YLGGLGFGWK
WNMGWMNDTL EYFKKDPVHR KYHHNELIFS ILYAFTESFV LSLSHDEVVH GKASLLTKMP GDEWQKFANL
RLLYGYMFTH PGKKLLFMGS EIAPWTEWHH EAGLEWHILD YERHAGIQRW VRDLNKAYCT LPALHQLDFS
EEGFSWIDCS DWENGVLSYS RKGFDPKDTA VVILNLTPVP RENYKIGVPF SGNWQEVLNS DSHYYGGSDK
GNFGRVASYP IAMHGHDYAL SLTLPPLAMI CLSPE

Supplementary Figure 6: a Native-PAGE containing glycogen reveals α-1,4 Glucanotransferase, elongation and hydrolytic activities of *E. lausannensis* GlgE. *E. coli* crude extract (CE) expressing GlgE-EL and purified GlgE-EL fraction (E1) were loaded onto native-PAGE containing 0.3% (w/v) of glycogen from bovine liver. Gel runs in ice pocket during 1h30 at 15 mA constant in TRIS/glycine buffer pH 8.8. After electrophoresis, native-gel was cut in three pieces and incubated overnight at room temperature with 10 mL 25 mM TRIS/acetate buffer pH 7.5 (Ø), 10 mL 25 mM TRIS/acetate buffer pH 7.5 and 1 mM of maltose-1-phosphate (M1P), 10 mL 25 mM TRIS/acetate buffer pH7.5 and 20 mM orthophosphate (Pi). Soaking native gel in iodine solution evidences GlgE activity. α-1,4 Glucanotransferase activity is visualized as brownish activity bands due to maltosyl reaction transfers catalyzed by GlgE-EL on the external glucan chains of glycogen particles. In presence of 1 mM M1P, the elongation activity is favored and consists in the maltosyl moieties transfer reactions of M1P onto non-reducing ends of external glucan chains of glycogen. The increase of long glucan chains leads to a strong iodine-glucan interaction observed as a dark activity band. At contrary, the hydrolytic reaction is conducted in the presence of 20 mM of Pi. GlgE-EL releases M1P from the non-reducing ends of external glucan chains of glycogen and α-1,6 linkages or branching points prevent the complete hydrolysis of glycogen particles.

Nevertheless, the resulting branched glucans escape from polyacrylamide gel leading to clear activity band in orange background. **b** Purification of branching enzyme (GlgB) activity of *Waddlia chondrophila*. The plasmid expression pET15b-GlgB-WC was transferred in ΔglgB Rosetta™ *E. coli* strain impaired in endogenous branching enzyme. After induction, crude extract (CE) was incubated for one hour with his-agarose beads at 4°C. Unbound proteins were eluted with 50 mM sodium acetate, 300 mM NaCl and 60 mM imidazole pH 7. After four washing steps, His-GlgB were eluted with 50 mM sodium acetate, 300 mM NaCl and 250 mM imidazole pH 7. Proteins in the flow through and elution fractions were separated on native-PAGE (7.5%) at 4°C (120 V, 15 mA). After electrophoresis, proteins were electrotransfered against a native-PAGE containing 0.3% (w/v) of potato starch using Trans-Blot® Turbo™ transfer system (Bio-Rad). Native-PAGE was then incubated overnight in 25 mM TRIS/acetate buffer pH 7.5 at room temperature. Branching enzyme activity is revealed as pink bands in blue background after soaking the gel in iodine solution (KI/I_2). **c** The uniprot accession number and amino acid sequence of recombinant proteins are displayed for GlgB protein. Amino acids underscored correspond to the His-tag and the att site produced during the cloning process in the expression vector.

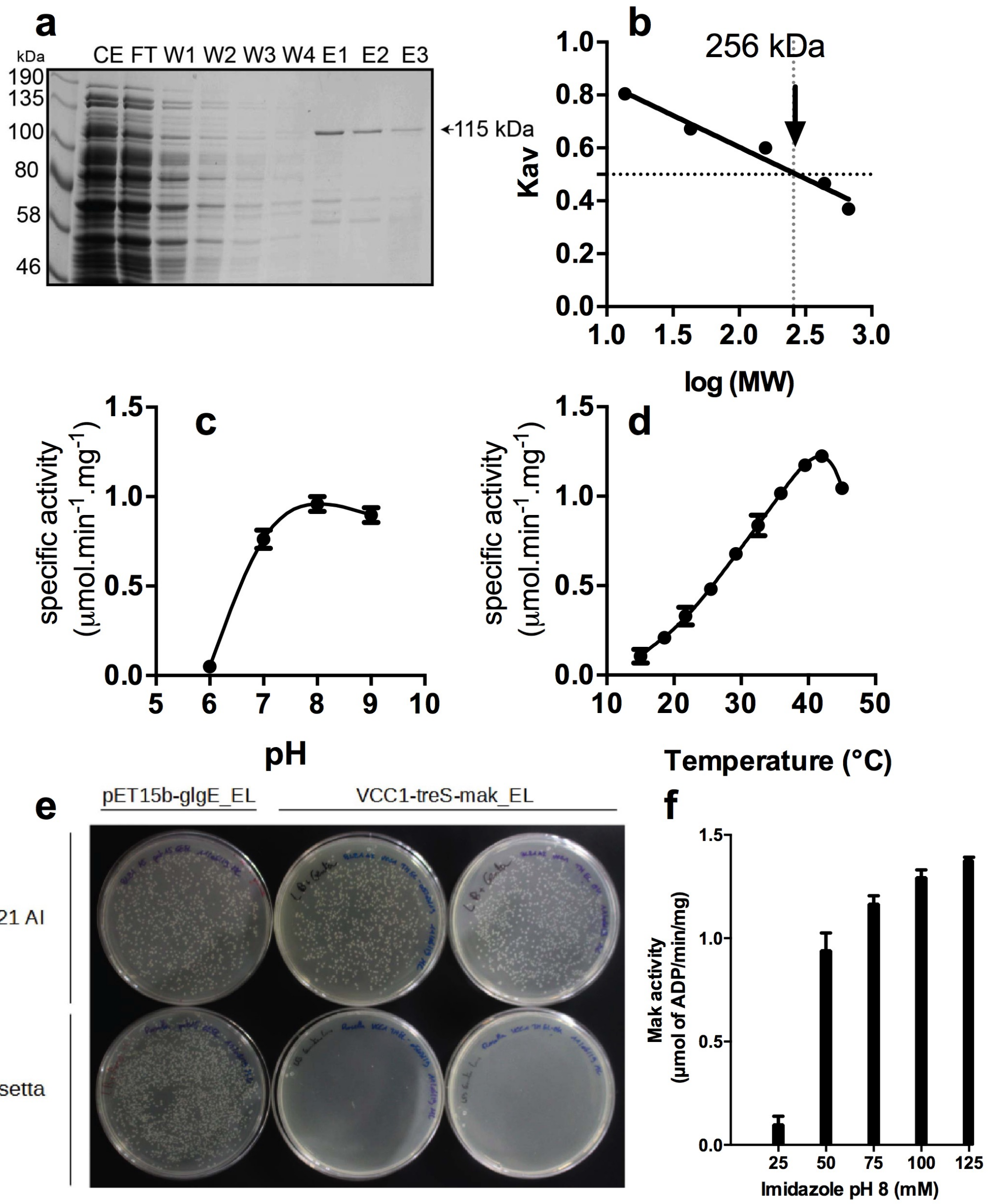

g **TreS-Mak_EL** Uniprot accession number : A0A0H5DQ88

MGSSHHHHHH SSGLVPRGSH MLEDHQTSLY KKAGFMKQDP LWFKHAVFYQ LHVKCFRDSN HDGIGDFRGL TEKLDHIQNL GCNAIWLQPF
YPSPLRDDGY DIADYHAIHP DYGTLKDFKL FLKEAKERGI RVVTELVLNH TSDQHAWFKR ARLSPKGSKW RNFYIWSEAP DKYLDCRIIF
KDFETSNWAF DPVADSYYFH RFYSHQPDLN YDNPEVHQEI FKIVDFWLGM GVDGLRLDAV PYLYKREGTD CENLPETHAF LKKLRSYIDS
KYENKMLLAE ANQWPEQACL YFGEGDECHM AFHFPVMPRL FMAIHKEDRF PIIDILDQTP TPPAVCQWAM FLRNHDELTL EMVTDEERDY
MWRVYAKDPR ARINLGIRRR LAPLMENDRQ KIELMFTLLL CLPGSPVIYY GDEIGMGDNY YLGDRNGVRT PMQWNNDRNA GFSDGDPQKL
YLPLIIDPKY HHAHVNVENE EQSPSSLLSW IKTVIAIRQA HPAFGSGTLK FVDAKNPKVL AFVREYDGDA ILVVVNLSRF SQYVEIDLAD
WKGSIPVDLF SHNNFANITA ADYGLTLGRY GYFWLALEPA KMRTSSHEEP IGHKFSLESS WDEVLKKENR TLMLKESLPQ FLSKQRWFRS
KGKPIKKVQI QDEVKFGNAK ILNVGVSFLD DQQENYQLPV TFIPASELTQ LKKEAPNSII AEIEVAGERG ALADAVYQKE FRTSLLDALI
RKSTMVSQSG EVFSESIAKL RKTFLDRKEV ESSLMTMEQS NTSINYGDSV ILKLYRRIEE GTNPDFELVR HLTEKSGFGH VPEYLGGIEW
KVGPSPKSQI ALAQRFIEKG TSAWDFTLQE METKFPELEE KTQEILKRSD SPERKRALVK AEAERVFKDY LKLADLLGER TAGLHLAFSS
DNESPEMVPE SFSMLYQKSL YQAMRTQTKK TLELLKERIS SLKPDEAELA RKVLHCEESI LTILKRLETR RFSAHKIRIH GDLHLGQIIC
TGSDALIIDF EGEPALPLGE RRLKRPVIQD IAGMVRSFDY ALTHFIISRG KTKNEAATSA KDALSLALRE RFTQSYLNAI KGAKTPLVPD
NPTELSILMN AFLLNKAIYE IGYEIRNRPD WIQIPMQGLL
NILKEMR

Supplementary Figure 7: **a** Recombinant his-tag TreS-Mak was purified on nickel affinity column and total protein of crude extract (CE), Flow through (FT), Washing steps (W1, W2, W3, W4) and Elution step (E1, E2, E3) were separated on 7.5% SDS-PAGE. Based in molecular weight standard, blue Coomassie staining revealed a polypeptide at

115 kD. **b** Superose 6 Increase 10-300 GL column (GE-Healthcare) pre-equilibrated with 140 mM NaCl, 10 mM orthophosphate pH 7.4 was calibrated with standard proteins (669; 440; 158; 43 and 13.7 kDa) and Blue Dextran. The determination of a partition coefficient (Kav) of 0.5 suggests an apparent molecular weight of 256 kD. **c** pH and **d** temperature optima of Mak activity were assayed with reaction buffer and same incubation time as described above. Mak activity was determined at pH 6, 7, 8 and 9 using imidazole (125 mM) as buffer at 30°C. Optimal temperature was inferred with reaction buffer containing 125 mM imidazole pH 7. Mean and standard deviation of three independent experiments are plotted. **e** Toxicity of TreS-Mak activity of *E. lausannensis***.** Two chemically competent *E. coli* cells Rosetta™ and BL21-AI™ were transformed with 0.15 µg of expression plasmids VCC1-TreS-Mak-EL (7.2 kpb) as well as with 0.15 µg of pET15-GlgE-EL (7.9 kpb) used as a control. Following the transformation, cells were spread onto Luria Broth medium containing appropriate antibiotic. Despite the lack of inductor (IPTG), no Rosetta™ colonies were observed after 16 hours at 37°C for two independent constructions of VCC1-TreS-Mak#1 and #2 while a close number of BL21-AI colonies are visualized with pET-15-GlgE and VCC1-TreS-Mak plasmids. The leaky transcriptional repression of LacI leads to the synthesis of TreS-Mak activity and *per se* the synthesis of highly toxic maltose-1-phosphate that do not occur in BL21-AI strains. **f** Effect of imidazole concentration on maltokinase activity of TreS-Mak. Maltokinase activity incubated in a reaction buffer containing maltose (20 mM), ATP (20 mM) Mn^{2+} (10 mM) and various concentrations of imidazole at pH 8 (25 mM to 125 mM). The production of ADP (n=3) is enzymatically determined after 40 minutes at 42°C (see material and methods). **g** The uniprot accession number and amino acid sequence of recombinant proteins are displayed for TreS-Mak protein. Amino acids underscored correspond to the His-tag and the att site produced during the cloning process in the expression vector.

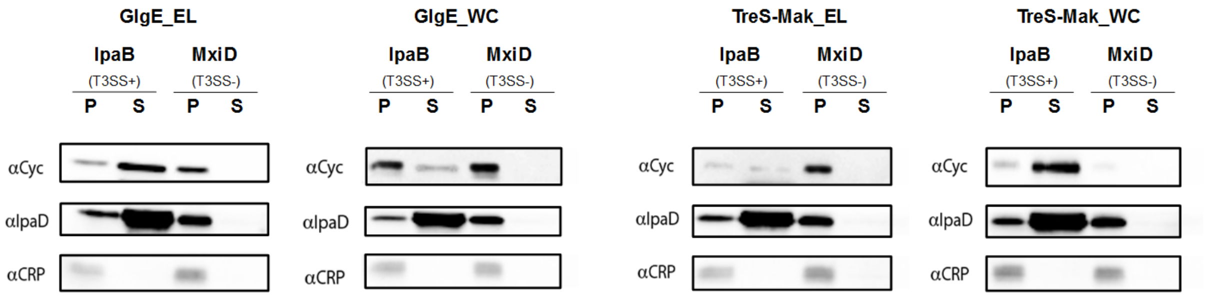

Supplementary Figure 8: **Heterologous secretion assay in *Shigella flexneri* of GlgE and TreS-Mak proteins.** The first thirty amino acids at N-terminus extremity of each protein were fused with the reporter protein adenylate cyclase from *B. pertussis* (Cyc). Fused proteins are expressed in IpaB (T3SS+) and MxiD (T3SS-) strains of *S. flexneri* harboring a functional and a defective type-three secretion system, respectively. Western blot analyses were performed on both cell pellets (P) and supernatants (S) using adenylate cyclase antibodies (αCyc). In parallel, a secreted (IpaD) and a non-secreted protein (CRP) were selected as positive and negative controls, respectively. Both proteins were detected in cell pellets or supernatants using αCRP and αIpaD antibodies. Those preliminary results suggest that both GlgE and TreS-Mak proteins of *Estrella lausannensis* (EL) and *Waddlia chondrophila* (WC) are secreted by the type-three secretion system.
