# Supplementary material for: Conservation of the glycogen metabolism pathway underlines a pivotal function of storage polysaccharides in Chlamydiae
Source: Commun Biol. 2021 Mar 5;4:296. doi: 10.1038/s42003-021-01794-y (PMC7935935; doi:10.1038/s42003-021-01794-y)
Supplement: Supplementary file 2 — Description of Additional Supplementary Files [file 42003_2021_1794_MOESM2_ESM.docx]

Description of Additional Supplementary Files

**Name**: Supplementary Data 1

**Description**: This Supplementary Data 1 is deposited in an external doi-minting repository: Dryad Digital Repository (<https://doi.org/10.5061/dryad.8sf7m0cm7>). This data file is composed of amino acid sequences used for phylogenies of both GlgE and TreS-MAK proteins. Inside, you will find the raw sequences, the aligned sequences as well as the blocks selected to perform phylogenies. Finally, the raw trees generated during the current study are provided for each of them. In addition, the GlgE phylogenetic tree performed with the same dataset under LG model with 1000 ultrafastbootstraps is provided as a raw tree and with a PDF version, as well.

**Name**: Supplementary Data 2

**Description**: This file contains spreadsheets that contain the original data used to determine the kinetic parameters and the enzymes properties displayed in the figures 5 and 8 in our manuscript.
